# Cyclical modulation of human ventricular repolarization by respiration

**DOI:** 10.3389/fphys.2012.00379

**Published:** 2012-09-24

**Authors:** Ben Hanson, Jaswinder Gill, David Western, Michael P. Gilbey, Julian Bostock, Mark R. Boyett, Henggui Zhang, Ruben Coronel, Peter Taggart

**Affiliations:** ^1^Department of Mechanical Engineering, University College LondonLondon, UK; ^2^Department of Cardiology, Guys and St. Thomas's Hospital and Kings College LondonLondon, UK; ^3^Department of Neuroscience, Physiology and Pharmacology, University College LondonLondon, UK; ^4^Division of Cardiovascular Medicine, University of ManchesterManchester, UK; ^5^Biological Physics Group, University of ManchesterManchester, UK; ^6^Experimental Cardiology Group, Academic Medical CenterAmsterdam, Netherlands; ^7^Neurocardiology Unit, University College London HospitalsLondon, UK

**Keywords:** respiration, cardiac electrophysiology

## Abstract

**Background:** Respiratory modulation of autonomic input to the sinus node results in cyclical modulation of heart rate, known as respiratory sinus arrhythmia (RSA). We hypothesized that the respiratory cycle may also exert cyclical modulation on ventricular repolarization, which may be separately measurable using local endocardial recordings. **Methods and Results:** The study included 16 subjects with normal ventricles undergoing routine clinical electrophysiological procedures for supraventricular arrhythmias. Unipolar electrograms were recorded from 10 right and 10 left ventricular endocardial sites. Breathing was voluntarily regulated at 5 fixed frequencies (6, 9, 12, 15, and 30 breaths per min) and heart rate was clamped by RV pacing. Activation-recovery intervals (ARI: a surrogate for APD) exhibited significant (*p* < 0.025) cyclical variation at the respiratory frequency in all subjects; ARI shortened with inspiration and lengthened with expiration. Peak-to-peak ARI variation ranged from 0–26 ms; the spatial pattern varied with subject. Arterial blood pressure also oscillated at the respiratory frequency (*p* < 0.025) and lagged behind respiration by between 1.5 s and 0.65 s from slowest to fastest breathing rates respectively. Systolic oscillation amplitude was significantly greater than diastolic (14 ± 5 vs. 8 ± 4 mm Hg ± SD, *p* < 0.001). **Conclusions:** Observations in humans with healthy ventricles using multiple left and right ventricular endocardial recordings showed that ARI action potential duration (APD) varied cyclically with respiration.

## Introduction

The timing of ventricular activation exhibits rhythmic cyclical variation with the respiratory cycle, whereby the interval between heartbeats increases and decreases with expiration and inspiration respectively: respiratory sinus arrhythmia (RSA) (Anrep et al., 1936). Although the mechanisms are complex (Anrep et al., 1936; Cohen and Taylor, 2002; Eckberg, 2009), RSA is generally agreed to result from waxing and waning of autonomic nerve input to the sinus node. We hypothesized that these mechanisms may also influence ventricular action potential repolarization. Unlike ventricular activation which is rapid, ventricular repolarization is very much slower and spatially heterogeneous, inscribing the T-wave of the ECG. Dynamic changes in ventricular action potential duration (APD), and hence repolarization time, are of great importance to fundamental electrophysiological mechanisms. Hence respiratory-induced fluctuations in APD might thereby play a role in a number of physiological and pathophysiological functions, including instances of sudden arrhythmic death attributable to sleep apnea.

We hypothesized that if APD exhibited localized oscillatory modulation with respiration, it might be observed in local electrical recordings from the *in-situ* human heart; our preliminary observations indicated the presence of a direct relationship (Gill et al., 2010). Here we report findings demonstrating respiratory-related cyclical changes in APD in human ventricles observed using multiple endocardial electrical recordings. Such cyclical changes were observed in conditions where both heart rate and respiratory rate were clamped.

## Methods

### Ethical approval

The study was approved by the Guy's and St. Thomas's Hospitals Ethics Committee and conformed to the standards set by the Declaration of Helsinki (latest revision: 59th WMA General Assembly). Informed consent was obtained in writing from all subjects.

### Subjects

Studies were performed in 16 patients (15 males, 1 female, age range 41–73, median 65 years), who were undergoing radiofrequency ablation procedures for supraventricular arrhythmias. 10 patients had established atrial fibrillation, 5 paroxysmal atrial fibrillation and 1 atypical atrial flutter. All subjects were otherwise healthy and had normal ventricular function. All studies were performed in the cardiac catherization suite at St. Thomas's Hospital and conducted prior to the routine clinical procedure in the un-sedated state, as previously described (Taggart et al., 2003; Hanson et al., 2009). Antiarrhythmic drugs and any other cardio-active medications were discontinued for 5 days prior to the study, which was sufficient wash-out time for all drugs in this population.

### Electrophysiological recording procedure

**Figure 1:**
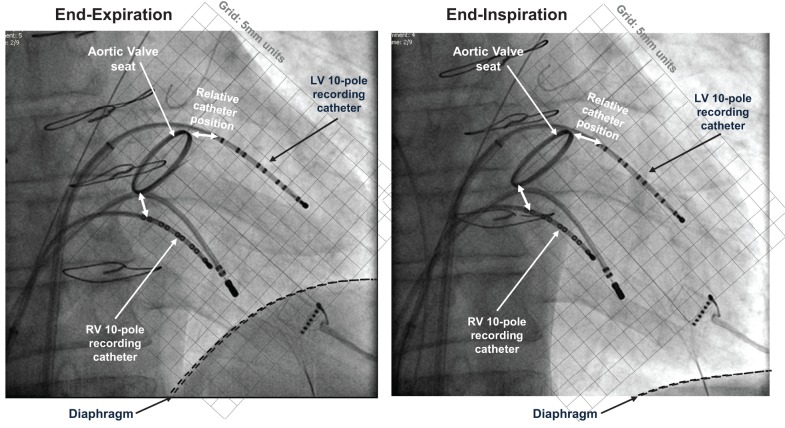
**Electrical recording methods.** Fluoroscopic images at end-expiration and end-inspiration of respiratory cycle, both at end-diastole. 10-pole electrode catheters are located in the left and right ventricles, with additional pacing catheter at right ventricular apex. This patient *(not included within the population of this study)* had a replacement aortic valve with radiopaque aortic ring; this and the sternal wires provide position markers by which to quantify relative positions.

One decapolar electrode catheter [St. Jude Medical (St. Paul, MN, USA) 6F Livewire™ Steerable Catheter model 401915 with 2-5-2 mm spacing, 35 mm total span] was introduced from the femoral vein, passed from right atrium to left atrium via a trans-septal puncture and across the mitral valve, and positioned in a base to apex orientation on the postero-inferior endocardial wall of the left ventricle. A second decapolar electrode catheter (as above) was introduced into the right ventricle from the femoral vein and across the tricuspid valve, and positioned in a base to apex orientation on the anterior septal wall. The reference electrode for all 20 unipolar electrograms from each subject was a large (100 × 150 mm) skin-surface electrode placed on the abdomen at approximately the level of the navel. Thus its relative distance to each individual electrode was considered to be approximately equal compared to the electrode inter-spacing (alternate 2 and 5 mm intervals). A pacing electrode was positioned at the right ventricular apex away from the decapolar recording electrode. Fluoroscopic cine-imaging was used to verify that the catheter tips maintained contact with the endocardium throughout the respiratory and cardiac cycles, Figure 1. Over the respiratory cycle relative motion is seen between the diaphragm, heart and sternum, but the recording catheters were observed to follow ventricular wall motion and retained their position to within 2 mm relative to the aortic root marker. No subject was known to have ventricular scar or have disordered conduction due to bundle branch abnormality. Arterial blood pressure was measured from the femoral artery using a continuous-flush pressure transducer system (TruWave PX600F, Edwards Lifesciences, Irvine, CA, USA).

### Controlled respiration

Subjects were briefed and asked to follow a controlled respiration protocol guided by a custom-created computer-generated animated visual display representing lung volume, which cycled at the required respiration rate (the display was implemented in LabVIEW software, National Instruments Corp., Austin, TX, USA). This was presented on a 19″-diagonal backlit LCD screen mounted directly in their natural line of sight while in a resting supine position. Subjects were instructed to breathe in a natural manner, taking deeper breaths at slower breathing rates than at faster breathing rates. The subject's breathing cycle was monitored using a custom-constructed tension sensor fixed to a freely-expandable band placed around the chest/abdomen (adapted from a RESPeRATE device, InterCure Inc., New York, NY, USA). The optimum location for each subject was chosen as the site of maximum circumferential strain during normal breathing. Tension in the elastic band was directly proportional to band circumference and this output was digitized and recorded at a sample frequency of 1200 Hz, synchronized to intracardiac electrograms and blood pressure recordings.

### Protocol

Pacing was established using a Biotronik (Berlin, Germany) model UHS 3000 stimulator, with an electrode positioned at the right ventricular apex, at a pulse width of 2 ms and stimulus strength of 2 × diastolic threshold at a minimum cycle length sufficient to maintain capture (median 500 ms). Following a 2 min period of adaptation to the paced cycle length the subject breathed at each frequency (6, 9, 12, 15, and 30 breaths per min) for 90 s each in random order. In a subset of 5 subjects blood gases were analyzed during normal breathing prior to controlled-rate breathing and on three occasions equally spaced throughout the protocol.

### Analysis of data

**Figure 2:**
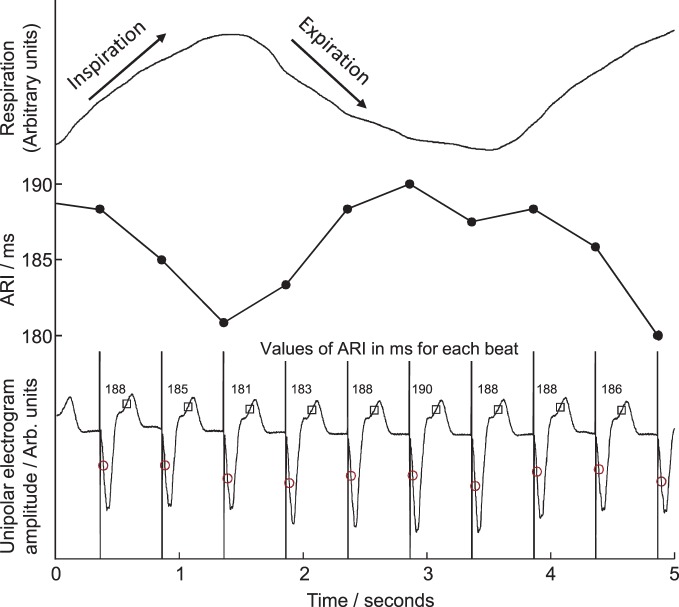
**Example of local unipolar electrograms recorded from an electrode site on the right ventricular endocardium.** Times of local activation are marked with circles, repolarization with squares, activation-repolarization intervals (ARIs) are labeled above each beat. ARIs are plotted in central panel for comparison with respiration trace, top, during fixed-rate breathing at 15 breaths/min. ARI shortened during inspiration, lengthened during expiration.

Twenty unipolar electrograms were obtained from the two decapolar catheters per subject, 10 from each ventricle, sampled and recorded at 1200 Hz (Ensite 3000, Endocardial Solutions Inc.) then exported for off-line analysis on a personal computer. At each recording site, activation-recovery intervals (ARI), as a surrogate for APD, were measured using the Wyatt method (validated in theoretical, computational and experimental studies (Wyatt et al., 1981; Millar et al., 1985; Haws and Lux, 1990; Coronel et al., 2006; Potse et al., 2009) where the moment of activation is taken as the moment of minimum dV/dt of the QRS complex of the unipolar electrogram (Wyatt et al., 1981; Millar et al., 1985; Haws and Lux, 1990; Coronel et al., 2006; Potse et al., 2009), and the moment of repolarization as the moment of maximum dV/dt of the T-wave of the local unipolar electrogram. The latter criterion is independent of the polarity of the T-wave. Example measurements are shown in Figure 2. Such measurements were computed using a semi-automated system which firstly applies a heuristic-based screening to identify and discount any cases where the T-wave is indistinct or corrupt, and then calculates the timing of activation and repolarization events using the Wyatt criteria, with further validity checks based on relative magnitudes of key deflections in the electrograms. The algorithm, including error-checking, is particularly designed for robust measures in the presence of noise; for full details see Western et al. (2010) (algorithm implemented using MATLAB R2012a, Mathworks, Inc., Natick, MA, USA). Systolic peaks and diastolic troughs in arterial blood pressure for each beat were measured from continuous traces of pressure from a femoral artery along with their times of occurrence.

### Statistical/numerical analysis

**Figure 3:**
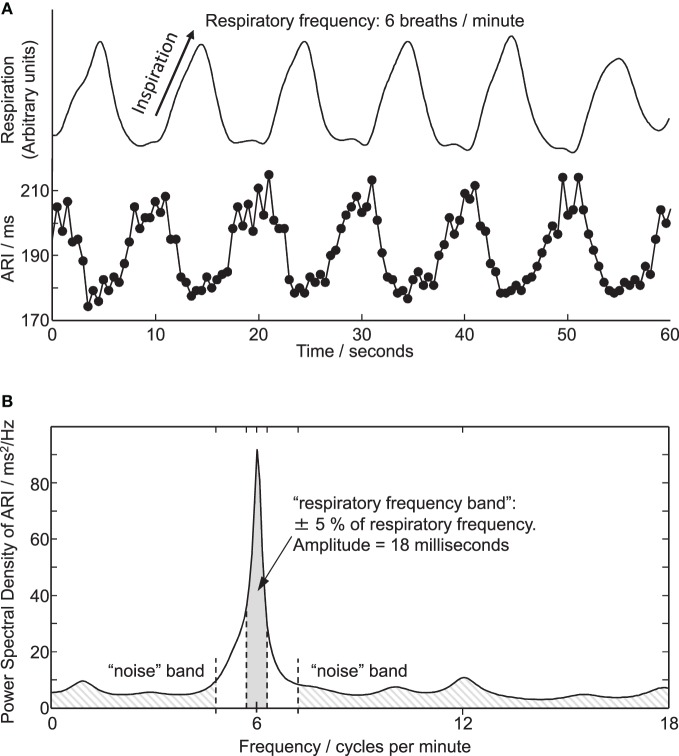
**(A)** Respiration and simultaneous ARI measures from an electrode site on LV endocardium showing cyclic variation at the respiratory frequency of 6 breaths/min. (**B**) Power spectral density (PSD) plot of the ARI measure computed over the entire 6 breaths/min recording. Peak in ARI oscillation coincides with respiratory frequency of 6 breaths/min. Shaded areas illustrate frequency bands used for calculating amplitude and statistical significance of oscillations, see “Methods” section.

The statistical significance of respiratory oscillations in ARI (Table 1) was determined using the following spectral method, programmed in MATLAB R2012a (as above). To establish an evenly-sampled series, any beats for which ARI measurement could not be determined were replaced by linear interpolation between the surrounding beats. If these surrogate beats constituted more than 10% of any series, the series was rejected. Otherwise, an auto-regressive Yule-Walker approach was applied, in preference to Fourier-Transform methods because it avoids the spectral resolution being constrained by a relatively short time series (90 s). Following the recommendation of Kay (1999), several model orders were tested and the optimal model order for each sequence was then chosen as that which minimized Akaike's Information Criterion (Akaike, 1974). A minimum order of L/3 (where L is the length of the series) was applied in order to provide consistency in the model order between different recordings from any particular subject. To ensure the models were un-biased the prediction error from each model was required to pass a whiteness test. The frequency spectrum of the series was then calculated from the coefficients of the optimal model (Takalo et al., 2005), providing a power-spectral-density (PSD) series, see Figure 3. The statistical significance of the respiratory component of this series was determined by comparing it with components outside the respiratory frequency: this “noise band” covered the normal range of natural respiratory frequencies (0.15–0.4 Hz) while excluding a band covering the frequency to be tested ±20%. The respiratory-frequency component (M) was identified as statistically significant when the following rule was satisfied:

$$\frac{\left(M-{\bar{M}}_{n}\right)}{\sigma_{n}}\geq 1.96$$

**Table 1:** **Data at example rate of 15 breaths/min including maximum peak-to-peak amplitudes of oscillations in ARI and BP**.

**Subject**	**Number of sites with significant (*p* < 0.025) ARI oscillation (total sites available for study)**	**Maximum peak to peak amplitude of ARI oscillation/ms (location of electrode)**	**Peak to peak amplitude of BP oscillation/mm Hg**		**Mean BP/mm Hg**	
			**Systolic**	**Diastolic**	**Systolic**	**Diastolic**
1	12 (18)	14 (RV 2)	21	10	140	85
2	20 (20)	8 (RV 1 and LV 6)	7	2	159	89
3	11 (16)	7 (LV 8)	8	4	180	119
4	14 (15)	20 (RV 5)	16	6	135	80
5	11 (14)	16 (RV 10)	15	7	121	82
6	13 (18)	18 (RV 6)	24	8	117	67
7	16 (16)	20 (LV 5)	22	11	129	90
8	11 (20)	7 (RV 6)	10	5	144	103
9	7 (12)	11 (LV 10)	20	17	148	100
10	8 (11)	6 (LV 2)	11	6	103	67
11	9 (14)	25 (LV 9)	18	7	166	92
12	6 (9)	19 (RV 2)	16	15	128	88
13	7 (11)	24 (RV 8)	10	8	125	74
14	12 (17)	26 (RV 1)	10	8	112	76
15	6 (19)	12 (RV 10)	12	6	114	70
16	10 (15)	7 (RV 1)	9	4	149	82
			*p* < 0.001			

**Figure 4:**
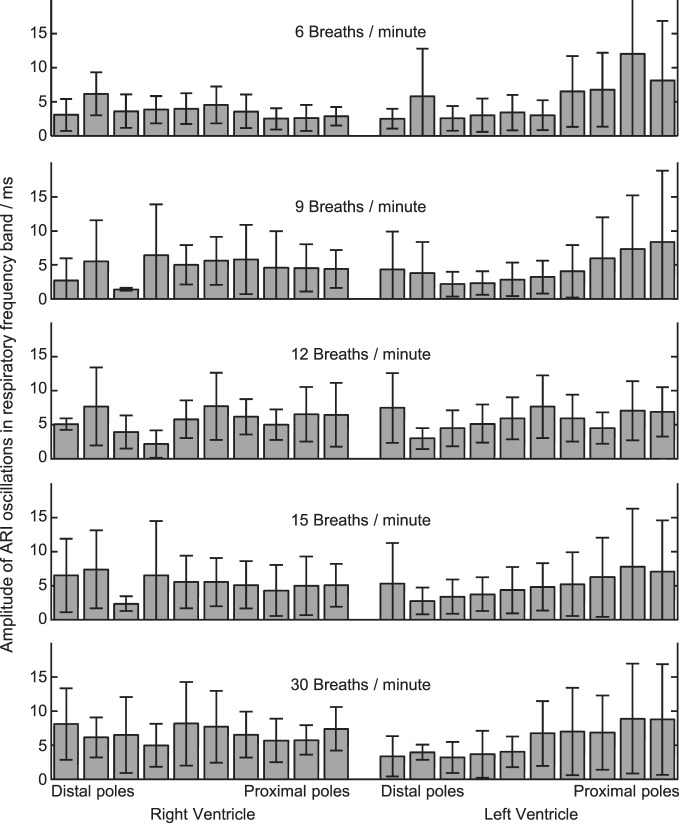
**Amplitude of ARI oscillations during constant-rate breathing at each of five rates (top to bottom).** Amplitude was calculated within the respiratory frequency band in each case, see “Methods” section and Figure 3. At each respiratory rate, ARI oscillation was measured at 20 local recording sites on the left and right ventricular myocardium. 16 subjects studied: mean ± standard deviation shown.

Location of electrode site given as Left or Right Ventricle (LV, RV) with catheter electrodes numbered 1–10, apex to base. Student's paired one-sided t-test was used to determine significance of difference in systolic vs. diastolic BP oscillations.

where ${\bar{M}}_{n}$ and σ_*n*_ are the mean and standard deviation of the spectrum in the noise band. The threshold value 1.96 was chosen to approximate a 97.5% one-sided confidence limit (*p* < 0.025). The magnitude of oscillations in ARI was quantified by integrating the area under the PSD plot between frequencies 5% either side of the respiratory frequency, as shown in Figure 3. This provided the oscillatory power in 2 ms; the square root of this value then provided the oscillation amplitude in milliseconds, as presented in Figures 3 and 4.

Table 1 provides further statistical data on ARI and BP at the example breathing rate of 15 breaths/min, including the maximum peak-to-peak values for ARI variation, measured for each subject. Significant differences between paired groups were determined using Student's *T*-Test. Mean values are reported ± standard deviation (SD).

Spectral analysis was used to demonstrate the magnitudes of oscillations and their central frequencies, however these linear spectral techniques are less well-suited to investigate phase relationships, since the waveforms are non-sinusoidal and change in shape over the frequency range studied (Karemaker, 2009). Instead cross-correlation was used (as before, MATLAB R2012a, Mathworks, Inc., Natick, MA, USA.) This process evaluates the similarity between two waveforms as one waveform is shifted in time relative to the other by increments, and identifies the time-shift “lag” in milliseconds associated with the maximum similarity (correlation). The respiration signal (tensile band) was cross-correlated with waveforms of ARI vs. time (up to 20 per subject) and systolic BP vs. time (one per subject). Only those waveforms showing significant (*p* < 0.025) oscillation amplitude were included. Cross-correlation was performed separately at each breathing rate, using the entire duration associated with that breathing rate. Lags were constrained within one respiration cycle period and are reported as absolute lags in milliseconds as well as the relative proportion of the respiratory cycle (0–360°).

## Results

A total number of 320 basic electrogram recordings were analyzed, obtained from 16 subjects. Results of blood gas analysis showed no significant variation with the exception of a slight reduction in PCO_2_ over the course of the protocol from median 4.7 to 4.1 kPa (*p* = 0.05, *n* = 5).

### Cyclical variation of ARI and BP with respiration

Cyclical variation of activation recovery intervals (ARI) was observed at the respiratory frequency. Figure 2 shows example unipolar electrograms exhibiting shortening of ARIs during inspiration, and lengthening of ARIs during expiration. In this example at 15 breaths/min one respiratory cycle lasts 4 s, during which the variation in ARI is 9 ms (5% of the mean ARI). The lengthening and shortening of ARI was cyclical at the respiratory frequency at each frequency tested: Figure 3A shows a further example at 6 breaths/min. The ARI exhibits some beat-to-beat variation but the most significant variation occurs at the respiratory frequency of 6 cycles per min. The magnitude of the variation at the respiratory frequency (and all other frequencies) was quantified by spectral analysis; Figure 3B shows the power spectral density (PSD) plot for the ARI waveform in Figure 3A, confirming the most significant oscillation coincides with the respiratory frequency. The “Amplitude,” calculated by integrating the area shown, represents the variation of ARI from the mean ARI occurring at the respiratory frequency. Since ARI exhibits variation at other frequencies, the total peak-to-peak variation recorded (Figure 3A) is greater than this value of amplitude at the respiratory frequency. Peak-to-peak ARI variations for each subject are reported in Table 1 at the example breathing rate of 15 breaths/min.

Statistical significance of oscillations was determined from comparing the relative magnitudes of the PSD at the respiratory frequency and the “noise band,” as illustrated in Figure 3B. Significant (*p* < 0.025) ARI oscillations were seen in all subjects but not at all electrode sites, and there was considerable inter-subject variability in their magnitude and spatial distribution (Table 1). There was no consistently significant Left-Right difference in the magnitude of oscillations across subjects however within the LV oscillation at distal recording sites (apical) was on average smaller than oscillation at proximal, more-basal sites. Figure 4 shows the oscillation amplitude at all recorded locations, at each breathing rate, averaged over all subjects. The magnitudes of ARI oscillation showed no significant variation between breathing rates. Tidal volume and volumetric air flow rates are likely to have varied across breathing rates, though unfortunately these were not quantified in this study.

**Figure 5:**
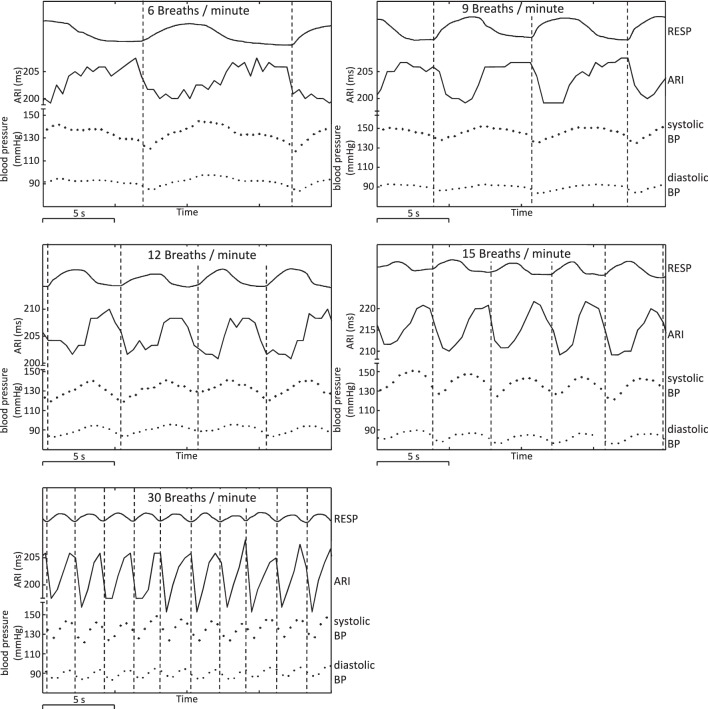
**Example plots showing respiration, ARI, and arterial blood pressure at respiratory frequencies of 6, 9, 12, 15, and 30 breaths/min, illustrating cyclical variation.** Arterial BP: systolic peak values and diastolic troughs for each heart beat are shown as individual points. Vertical lines have been added at the times of onset of inspiration to illustrate chronological (phase) relationships.

Oscillations in arterial blood pressure were observed in all subjects at the respiratory frequency. The amplitude of oscillation in the systolic pressure measures was significantly larger than the amplitude of oscillation in diastolic measures (14 ± 5 vs. 8 ± 4 mm Hg, mean ± SD, *p* < 0.001, Table 1). Figure 5 presents data from an example subject and shows cyclical variation of arterial blood pressure together with respiration and ARI. Similar effects were noted at each breathing rate tested, with BP and ARI oscillations matching the respiration frequency.

At each respiration frequency, inspiration was accompanied by a shortening of ARI, with expiration corresponding to a lengthening of ARI. Vertical lines on Figure 5 indicate onset of inspiration as measured from the chest circumference, and provide a guide to the chronological sequence of variation: in this example, shortening or lengthening in ARI commenced between 0.5 and 1 s prior to the onset of measured inspiration or expiration, with similar relationships between peaks in ARI and troughs in respiration, and vice-versa. Systolic arterial blood pressure reached a nadir shortly after onset of inspiration (interval 0–1 s in this example). Systolic BP increased during inspiration then reached a peak shortly after end-inspiration at maximum chest circumference and prior to onset of expiration. Diastolic measures of arterial BP followed the same pattern as systolic variation, but lagged behind on a beat-by-beat basis: increases and decreases in systolic BP were followed by corresponding increases and decreases in the successive diastolic measure.

**Figure 6:**
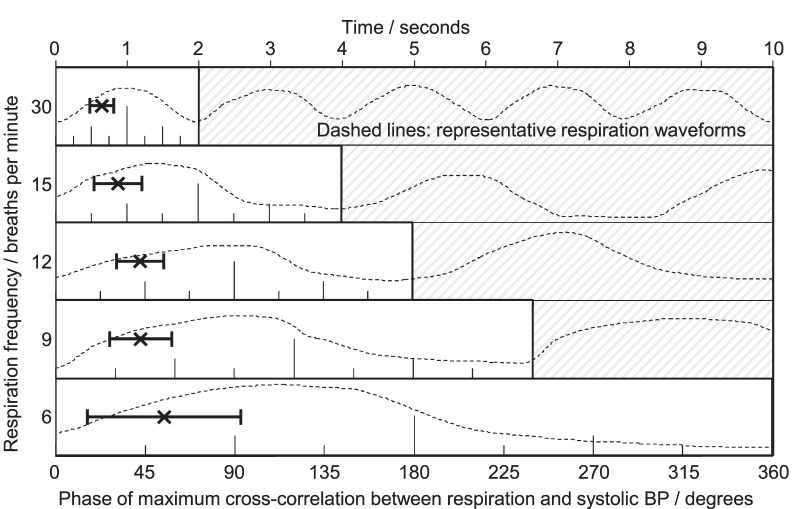
**Phase relationships obtained from cross-correlation between respiration and systolic blood pressure.** Five respiration frequencies are presented (top to bottom) on a common horizontal time axis; typical respiration waveforms are plotted against time as a guide. At each rate, one full respiratory cycle is marked in degrees (0–360°). Each plot shows the mean ± SD lag for all subjects, readable as absolute lag in seconds (top scale) or relative lag in degrees of one full cycle (individual scales). BP fluctuations lagged behind respiration at all respiratory rates. The mean lag decreased slightly in absolute terms from 1.5 s at the slowest rate of 6 breaths per min to 0.65 s at the fastest rate of 30 breaths per min. As proportions of the respiratory cycle, the lags increased from 55 to 116°, respectively.

Cross-correlation was used to quantify the phase differences between cyclical variation in the respiration signal (chest circumference) and BP across all breathing rates and subjects. Figure 6 shows the relationship between respiration and systolic BP: in every case BP variation lagged the respiration signal, by between 0.3 and 2.1 s overall. The mean lag decreased as the respiratory cycle duration decreased (as the breathing rate increased from 6 to 30 breaths/min). The relationship was not in proportion to the respiratory cycle; tick marks on the x-axis of each breathing rate plot indicate the relative phase of the respiratory cycle in degrees and show that the relative lag increases monotonically from 55° at 6 breaths/min to 116° at 30 breaths/min.

**Figure 7:**
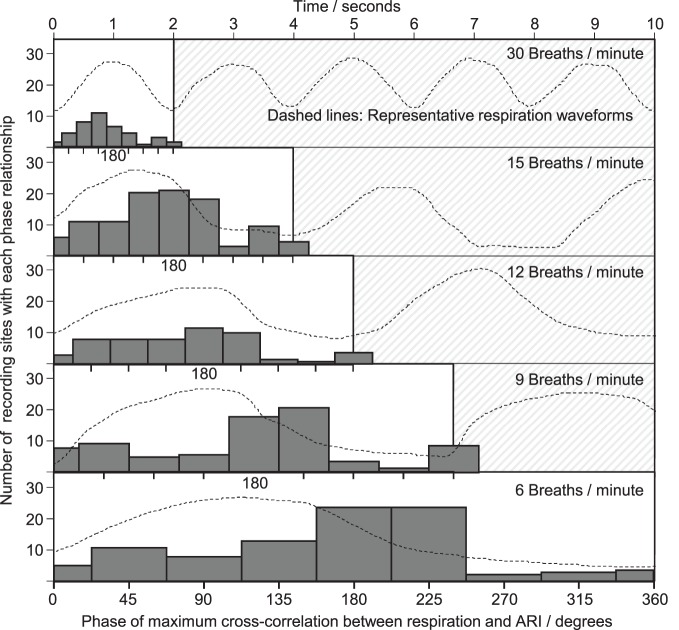
**Phase relationships obtained from cross-correlation between respiration and ARI.** As with Figure 6, a common horizontal time axis is used, and typical respiratory waveforms are shown, with one cycle highlighted. At each respiratory rate, one cycle is divided into 9 ranges (with 0 and 360° shown separately) and a histogram collates the phase lags computed at each electrical recording site within each subject. The height of each bar represents the total number of electrical recording sites with a maximum cross-correlation lag falling within that phase of the cycle. The most frequently occurring phase lags were clustered around 180° between respiration and ARI.

Cross-correlation was performed between the respiration signal and every measure of ARI across all recording sites, breathing rates and subjects, which passed screening for validity, and which exhibited significant oscillations at the respiratory frequency. The histogram results in Figure 7 exhibit a spread in the range of identified lags between respiration and ARI with a peak occurring approximately halfway through the respiratory cycle. As the respiratory rate increased, the time-lag between respiration and ARI measures decreased proportionally with the decrease in respiratory cycle duration, such that at each rate the maximum correlations occurred most frequently around 180° phase difference: shortest ARIs occurred with maximum respiration measures (maximum chest volume). This relationship was consistent at each respiratory rate with the exception of 30 breaths per min when the peak correlations occurred at a slightly shorter lag, around 135° of the cycle.

## Discussion

Using electrical recordings obtained directly from normal ventricles at a constant paced rate in conscious humans, these results show that ARI exhibited cyclical fluctuations with breathing over breathing frequencies from 6 to 30 breaths/min. This indicates that ventricular APD varies with the breathing cycle, independently of heart rate variation.

### Experimental model

Breathing strongly influences hemodynamics and is associated with rhythmic changes in sympathetic and parasympathetic activities controlling cardiovascular function (Eckberg, 2003). The combination of these factors causes variation of the sinus rhythm such that both heart rate and blood pressure rise and fall at the frequency of breathing (Larsen et al., 2010). In contrast, the experimental protocol here employed RV pacing in order to remove heart rate variability thus allowing controlled investigation of the effect of respiration on APD.

Extensive studies and clinical validations have demonstrated that the unipolar signal recorded at each pole along multipolar catheters such as used here (St. Jude Medical, St. Paul, MN, USA) is a true representation of the local endocardial activity, and the derived ARI measure is representative of the local APD (Wyatt et al., 1981; Millar et al., 1985; Haws and Lux, 1990; Coronel et al., 2006; Potse et al., 2009). Studies have demonstrated that catheters maintain location through the cardiac cycle, with standard deviation of typically 1 mm amplitude (Lessick et al., 2001). Our measurements of catheter positions through the respiratory and cardiac cycles confirmed these results; the catheters were observed to follow the ventricular wall motion through the cardiac cycle but there was no significant translation of the catheters along their axial direction through the respiratory cycle (see Figure 1). The catheters measured local unipolar electrograms along two 35 mm-long endocardial paths, which provided evidence of ARI variation with spatial heterogeneity. Heterogeneity over the entire myocardium is expected to be greater; global mapping is recommended for future study to further quantify patterns of spatial variation and whether there exist consistent patterns across the human heart.

Respiratory frequency was well-controlled by the subjects, however the experimental protocol did not control for lung volume, nor for intra-thoracic pressure fluctuation, due to the additional interference imposed by respiratory tidal volume and gas composition monitors. For this study, respiration was required to be voluntarily controlled, and the tidal change voluntarily adjusted to suit the wide range of rates, thus avoiding hyper or hypo-ventilation.

### Mechanisms

This study provides evidence of ARI and BP oscillation related to respiration, however while the phase analysis results demonstrated correlation that does not imply causation. Further—more invasive—study is necessary to determine the underlying mechanisms behind these observations. Here, we briefly discuss several possibilities.

### Haemodynamics

The breathing cycle is accompanied by intrathoracic pressure changes (Innes et al., 1993) which would impose directly on venous and arterial blood pressure recordings. However, oscillating intrathoracic pressure cannot be held wholly responsible for the oscillations observed here since BP measures showed a significant difference in amplitude between systolic and diastolic variation, which would not be the case if intrathoracic pressure variation was simply superimposed onto the measured blood pressure signal. This assertion is further confirmed by the result that the phase relationships between respiration and BP varied at different respiratory frequencies (Figure 6). Finally, the phase of BP oscillation was inconsistent with the intrathoracic pressure variation that would be expected with natural respiration (here, pressure minima occurred at minimum chest volume).

BP oscillation at the respiratory frequency could therefore result either from variation in cardiac function, or variation in afterload attributable to vascular tone. Since vasomotor dynamics would be unlikely to respond at sufficient speed to match the respiratory rates in this study (Julien et al., 2001) oscillation in cardiac mechanical function at the respiratory rate would seem the more likely explanation.

### Mechanical-electrical coupling

A possible mechanism for fluctuations of ventricular ARI at the respiratory frequency could be mechano-electric feedback whereby changes in ventricular loading alter the electrophysiology (Kohl et al., 2006; Taggart and Sutton, 2011). Respiration induces cyclical changes in ventricular pressure/volume relations with a reduction of ventricular filling occurring during inspiration (Innes et al., 1993). We have previously shown in humans using a model of instituting and discontinuing cardiopulmonary bypass in patients undergoing cardiac surgical procedures, that reducing ventricular filling and volume increased APD of epicardial monophasic action potentials (Taggart and Sutton, 2011). Conversely refilling the heart from a reduced volume load shortened APD. These findings are in keeping with a substantial amount work in a wide range of experimental models (Kohl et al., 2006; Taggart and Sutton, 2011). The direction of APD changes in relation to ventricular volume changes in these previous surgical studies is opposite to the ARI changes we observed with respiration. However, whether APD lengthens or shortens in response to mechanical stretch or deformation is critically dependent on the nature and timing of the mechanical perturbation, and therefore mechano-electric feedback remains a possible mechanism.

### Nervous mechanisms

Two potential mechanisms proposed to account for respiratory-related oscillations of sinus node firing (i.e., RSA) have been the subject of extensive investigation and ongoing debate (Task force, 1996; Malliani, 2000; Cohen and Taylor, 2002; Eckberg, 2009; Karemaker, 2009). Both mechanisms could be operative. Of these, the baroreflex mechanism posits that fluctuations in intrathoracic pressure synchronous with respiration induce changes in stroke volume (Innes et al., 1993) and hence fluctuations in arterial pressure. These rhythmic fluctuations in systemic arterial pressure are sensed by baroreflex afferent nerves resulting in appropriately increasing and decreasing efferent vagal activity, which induces corresponding fluctuations RR interval (De Boer et al., 1987). Fluctuations in arterial BP were observed in this study, therefore the baroreflex may have been generating fluctuating nervous activity at the respiration frequency. The results of phase analysis (Figures 6 and 7) over a range of respiration rates (6–30 breaths/min), indicate that this mechanism may partially—but not fully—explain the observed oscillations, as follows: the baroreflex is characterized by a delay (latency) in responding to arterial pressure fluctuations of at least one heartbeat, and up to 2.5 s (Julien, 2006). This time delay would produce a phase lag that would become much more significant at faster breathing rates; e.g., at 6 breaths/min, 2.5 s corresponds to ¼ of the cycle period (90°), but at 15 breaths/min 2.5 s is more than half the cycle period (>180°). Thus a baroreflex-driven model of interaction between BP and ARI would have a phase relationship which varied proportionally with respiratory frequency. Figure 6 shows that the relative phase lag between respiration and BP did indeed increase with increasing respiratory frequency (from 55 to 116°), however this was not consistent with the time lags associated with the baroreflex: the mean absolute lag was not constant but decreased from 1.5 to 0.65 s from the slowest to the fastest breathing rate. This suggests that the baroreflex mechanism did not have a major role in causing the ARI and BP oscillations observed here. An alternative mechanism to that of the baroreflex is central gating of autonomic drive to the heart by central respiratory networks (Gilbey and Spyer, 1997; Gilbey, 2004). Our results are consistent with the presence of fluctuating autonomic neural traffic to the ventricular myocardium sculptured by central respiratory activity, which arises through brainstem interactions (Spyer and Gilbey, 1988; Dergacheva et al., 2010) or through entrainment by activity arising in the cerebral cortex during controlled breathing (Evans, 2010). Interactions associated with the cardiac plexus may also be involved (Armour and Hopkins, 1990).

It is likely that the time delay for sympathetic nerve transmission at neuro-effector junctions would induce a degree of buffering of the response to cyclical neural input. During inspiration, there is also a decrease in parasympathetic activity (Kollai and Koizumi, 1981; Gilbey et al., 1984). Parasympathetic stimulation, via the release of acetylcholine (ACh), was thought to have no direct effect on the ventricles. However, it has been shown that, in mammalian ventricular myocytes (including human), ACh can activate the ACh-activated K+ current, IK, ACh, and shorten the action potential, (Koumi and Wasserstrom, 1994; Yang et al., 1996; Dobrzynski et al., 2002). Via this pathway, the decrease in parasympathetic activity during inspiration is expected to increase (rather than decrease) the APD (ARI). However, in addition, there can be an interaction between the effects of the two branches of the autonomic nervous system on the heart, a process known as “accentuated antagonism.” Parasympathetic stimulation can antagonize, both presynaptically and postsynaptically, the effects of sympathetic stimulation (Stramba-Badiale et al., 1991). Parasympathetic stimulation can inhibit the activation of ICa, L, and IK's caused by sympathetic stimulation (Nakajima et al., 1990; Freeman and Kass, 1995). Via this pathway, the decrease in parasympathetic activity during inspiration is expected to shorten the action potential.

### Implications

These endocardial data are, to the authors' knowledge, the first indication of alteration of APD attributable to respiration and independent of heart rate. The cyclical modulation of APD may play a role in a range of electrophysiological functions including arrhythmogenesis, in-keeping with the concept of a dynamic substrate created by the collective interaction of several dynamic physiological processes.

Our data support the idea that respiratory patterning of autonomic nervous activity influences repolarization by enhancing autonomic influences on cardiac electrophysiology (Gilbey, 2004). This action would be particularly robust in regions of high innervation, thereby potentially increasing electrophysiological inhomogeneity: autonomic nerve innervation is highly inhomogeneous, particularly in diseased hearts subject to nerve damage and regrowth, “nerve sprouting” (Chen et al., 2001).

The interaction between respiration and APD may be relevant to the well-known arrhythmogenic potential of sleep apnea and its associated irregular respiratory patterns, particularly in diseased hearts.

### Limitations

It has long been known that hyperventilation may induce repolarization changes in the electrocardiogram of normal subjects, for example T-wave flattening or inversion has been reported in up to 50–70% of healthy individuals (Bieberman et al., 1971), however these changes occur at a much slower rate than the breath-to-breath oscillations we observed here. Similarly, small changes in pCO_2_ were observed during the course of the fixed-rate breathing protocol. These changes may have affected pH and ion channel function and thereby APD and ARI. However, these changes occur in the course of h, whereas we studied rapidly occurring respiration dependent changes. Therefore, pH changes are not likely to have affected the results.

## Author contributions

Ben Hanson, Peter Taggart, Jaswinder Gill and Julian Bostock conceived and designed the experiments. All authors took responsibility in collecting, analyzing and interpreting the data, with particular individual input in the following areas: nervous control of cardiovascular and respiratory systems (Michael P. Gilbey), cellular mechanisms (Henggui Zhang and Mark R. Boyett), electrophysiology (Peter Taggart). All authors contributed to drafting or revising the manuscript and all authors approved the final version of the manuscript.

### Conflict of interest statement

The authors declare that the research was conducted in the absence of any commercial or financial relationships that could be construed as a potential conflict of interest.

## Abbreviations

APD: action potential duration
ARI: activation-recovery interval
LV: left ventricle
PCO_2_: carbon dioxide partial pressure
RSA: respiratory sinus arrhythmia
RV: right ventricle.
